# Comparative midterm results of surgical mitral valve replacement and transcatheter mitral valve replacement with the Tendyne System

**DOI:** 10.1016/j.xjse.2025.100045

**Published:** 2025-01-30

**Authors:** Andrea Colli, Cristina Di Franco, Federico Giorgi, Giosuè Falcetta, Danilo Ruggiero, Michele Celiento, Giacomo Ravenni, Riccardo Morganti, Laura Besola

**Affiliations:** a Cardiac Surgery Division, and Department of Surgical, Medical and Molecular Pathology and Critical Care, University of Pisa, Pisa, Italy; b Biostatistics Unit, Pisa University Hospital, Pisa, Italy

**Keywords:** mitral valve disease, TMVR, SMVR, Tendyne

## Abstract

**Objectives:**

To compare the midterm clinical and echocardiographic outcomes of high-risk patients who underwent surgical mitral valve replacement (SMVR) or transcatheter mitral valve replacement (TMVR) with the Tendyne System in a single-center setting.

**Methods:**

The study included all consecutive high-risk patients who underwent TMVR or SMVR at the University of Pisa, Pisa, Italy, between March 2018 and December 2023. The primary outcome was cardiovascular (CV) mortality at follow-up (FU), whereas secondary outcomes were overall mortality and rehospitalization for heart failure at FU.

**Results:**

We included 30 patients who underwent TMVR and 50 who underwent SMVR. Although the European System for Cardiac Operative Risk Evaluation II and Society of Thoracic Surgeons scores were similar, patients who underwent TMVR were older and had worse renal function and left ventricular ejection fraction (LVEF, 41.80 ± 13% and 56.89 ± 10.23%). The 30-day CV mortality was lower in the TMVR group (*P* = .055). Over a median FU of 1.9 (0.8-3.4) years, the overall mortality was 37.5% and 50% in the SMVR and TMVR groups, respectively (*P* = .277), whereas CV mortality was 25% and 33%, respectively (*P* = .496). New hospitalizations for heart failure were similar between the 2 groups (*P* = .352). Univariate analysis showed no impact of procedure type on CV (hazard ratio, 1.57; 95% confidence interval, 0.66-3.73, *P* = .311) and overall survival (hazard ratio, 1.67, 95% confidence interval, 0.82-3.41, *P* = .157). Mild paravalvular leak rate was low in both groups at discharge and FU. In both groups, the mean transmitral gradient was low at discharge and significantly decreased in the TMVR group during FU. In the TMVR group, LVEF did not change after the procedure (Δ pre-post −16 ± 28% *P* = .111) and then remained stable at FU (Δ post-FU –0.09 ± 17%, *P* = .695), whereas in the SMVR group, LVEF decreased after surgery (Δ pre-post 7 ± 19%, *P* = .031) to slightly improve over FU (Δ post-FU 7 ± 21%, *P* = .202). Right ventricular function did not change at any point in either group. In the SMVR group, patients experienced more tricuspid regurgitation worsening than those in the TMVR group (42% vs 15%, *P* = .101).

**Conclusions:**

SMVR and TMVR showed similar early and midterm clinical and echocardiographic outcomes in a high-risk population. However, TMVR resulted in a lower postoperative complication rate and faster recovery. Therefore, TMVR might be a possible alternative to surgery for selected high-risk patients.

**Figure:**
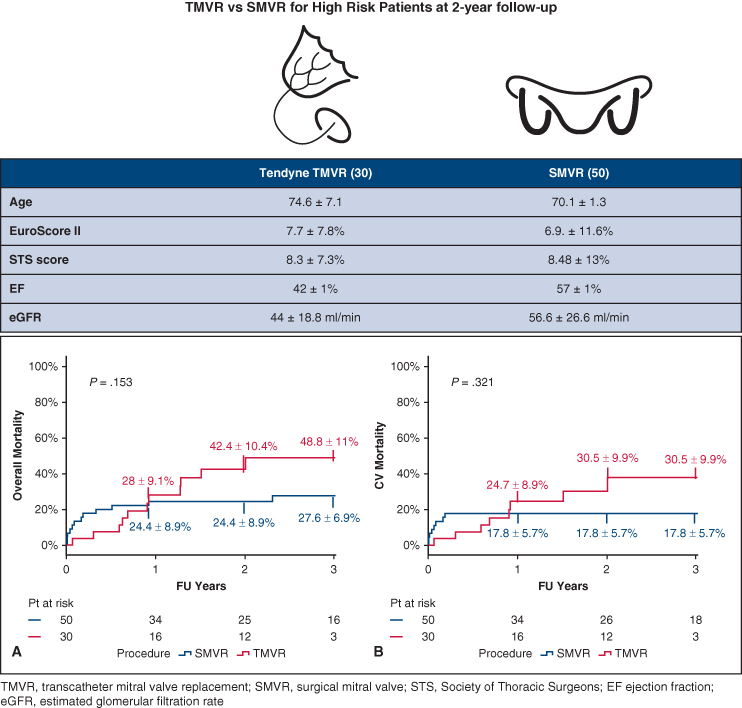
Risk profile and Kaplan-Meier curves of cumulative overall and cardiovascular mortality.

**Central Message**

In a cohort of high-risk patients with TMVR, the Tendyne System achieved clinical and echocardiographic outcomes comparable with SMVR at midterm follow-up.

**Perspective**

Compared with surgical MVR, TMVR demonstrated its effectiveness in high-risk patients, with shorter hospitalization time, acceptable survival, and improvement of functional status at early and midterm follow-up. It may therefore become a valuable option in patients who are not eligible for surgery or transcatheter repair also thanks to low transvalvular gradients and stable reduction of MR.

International guidelines recommend surgery for severe mitral disease, including mitral regurgitation (MR) and mitral stenosis (MS), in the presence of symptoms or echocardiographic signs of left ventricular (LV) dysfunction.^1^^,^^2^ Surgical mitral valve (MV) repair is the preferred treatment for MR, whereas surgical mitral valve replacement (SMVR) is performed when repair is not feasible.^1^^,^^2^ SMVR might be preferred over surgical MV repair for treating functional MR because of the reported lower rate of recurrent MR at 1- and 2-year follow-up (FU). Still, it is associated with greater operative mortality.^3^^,^^4^ New transcatheter techniques have been developed to improve outcomes and treat patients currently rejected for surgery.^5^^,^^6^ Transcatheter edge-to-edge repair has gained widespread adoption, but its feasibility heavily depends on mitral valve anatomical characteristics. Transcatheter mitral valve replacement (TMVR) is a proposed alternative. Among the devices under development or investigation, the Tendyne System (Abbott Vascular) is the only one that received Conformite Europeenne (CE) mark in 2020. The Global Feasibility Study and Expanded Clinical Study showed Tendyne's procedural safety and effectiveness in a 30-day and 2-year FU.^7^^,^^8^

Postmarketing independent registries are also recruiting off-label patients, such as those with previous MV intervention or surgery, mitral annular calcification, or left ventricular ejection fraction (LVEF) below 30%, and have confirmed encouraging early and midterm outcomes.^9^, ^10^, ^11^, ^12^ The role of TMVR in a real-world setting is still being established, and there is limited comparative data with SMVR.^13^ This study aims to compare the midterm clinical and echocardiographic outcomes of high-risk patients who underwent SMVR or TMVR with the Tendyne System at our Institution.

## Methods

### Study Design and Population

This was a retrospective observational study conducted at a single center. The study included only patients at least at intermediate risk (European System for Cardiac Operative Risk Evaluation [EuroSCORE] II ≥4%) affected by MR or MS who, between March 2018 and December 2023, underwent isolated SMVR or TMVR with the Tendyne System. Because this retrospective study focused on 2 standard surgical procedures, no institutional review board approval was required.

**Figure 1:**
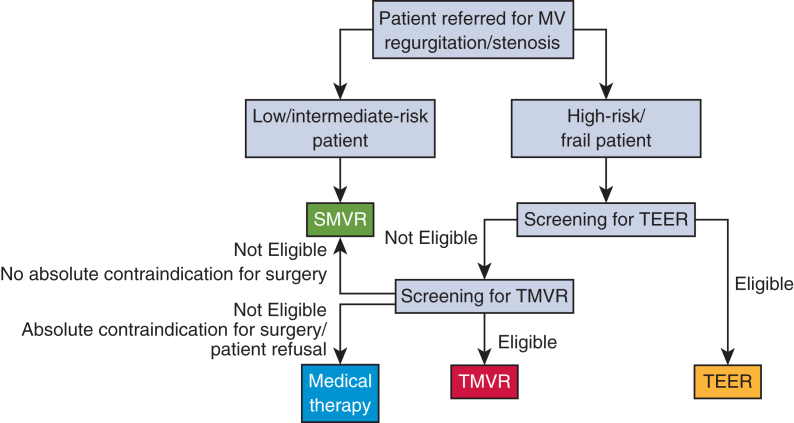
Flowchart showing the selection process used at our center. *MV*, Mitral valve; *SMVR*, surgical mitral valve replacement; *TEER*, transcatheter edge-to-edge repair; *TMVR*, transcatheter mitral valve replacement.

Patients were initially screened for SMVR. If they were deemed ineligible because of high surgical risk or contraindications for surgery, a Heart Team evaluation was performed. First, transcatheter edge-to-edge repair feasibility was evaluated, and if not feasible, TMVR screening was assessed. Patients with off-label indications, such as severe mitral annular calcification graded according to Guerrero's classification,^14^ severe MS, and redo MV procedures, were also included in the study. When patients were rejected also for TMVR, or it was not possible to complete the TMVR screening because of patient's hemodynamic instability, SMVR was performed instead unless in case of absolute contraindication for surgery (real porcelain aorta, never observed). Figure 1 shows the selection process.

Baseline, procedural, and postoperative data were collected by reviewing clinical charts. Data were collected and anonymized in accordance with the Declaration of Helsinki and European GDPR after obtaining patients’ written consent.

FU data, including clinical and echocardiographic information, were obtained through review of medical charts, outpatient clinic visits, and phone calls when not available otherwise. Echocardiographic FU was conducted in accordance with hospital policy. Both groups underwent similar follow-up at 6 months and yearly thereafter as standard outpatient's clinic visits, that included physical examination and 2-dimensional TTE.

Study primary outcome was cardiovascular (CV) survival at latest FU, and secondary outcomes included overall survival and rehospitalization for heart failure (HF) at latest FU. Baseline variables and postoperative complications were defined according to Mitral Valve Academic Research Consortium criteria.^15^

### Procedure

Surgical and transcatheter procedures were performed according to Center's policy. SMVR was performed in a standard operating room after the induction of general anesthesia and orotracheal (OT) intubation, with full cardiopulmonary bypass support and cardioplegic arrest. Surgical access, cardiopulmonary bypass conduction, and cardioplegic solution were performed according to surgeons' preferences. All types of commercially available biological and mechanical prostheses were accepted for SMVR.

Tendyne System implantation was performed with the patient under general anesthesia and OT intubation through a left lateral minithoracotomy as previously described.^16^ All SMVR and TMVR procedures were performed by the same experienced surgeon.

### Statistical Analysis

Continuous variables are expressed as mean (standard deviation) when customarily distributed and otherwise as median and interquartile range. Categorical variables are presented as frequencies and percentages otherwise. Continuous parameters were compared using the paired Student *t* test or 2-tailed Student *t* test. Categorical variables were compared using the χ^2^ test. Concordance analysis for paired categorical variables was performed using the kappa Cohen method. Factors influencing overall and CV survival were assessed with the univariate and multivariate Cox models weighted for procedure type. Cumulative survival was tested using Kaplan-Meier analysis with the log-rank test. All statistical tests were 2-sided. Analysis was performed using SPSS Statistics 29 (IBM Corp).

## Results

We identified 80 consecutive patients, 50 (62.5%) of who underwent SMVR and 30 (37.5%) TMVR. Patients rejected for SMVR and who were screened for TMVR in study period were 44, 8 patients were excluded for elevate risk of LV outflow tract obstruction, whereas 6 had an out-of-range anulus diameter (total rejection rate 31.8%); 5 of these patients were not offered SMVR and were medically treated (no FU data available), whereas the remaining 9 underwent high-risk SMVR in accordance with our ethical policy.

The patients who underwent TMVR were older (74.63 ± 7.16 years compared with 70.10 ± 10.33 years for SMVR patients) and had worse kidney function (estimated glomerular filtration rate [eGFR] 44.98 ± 18.81 mL/min compared with 56.60 ± 26.64 mL/min for patients who underwent SMVR). The majority of patients were symptomatic, but patients who underwent TMVR experienced a greater number of HF rehospitalizations before the procedure (73.3%), and nearly 50% of them had an implantable cardiac defibrillator or cardiac resynchronization therapy pacemaker. The 2 groups had similar EuroSCORE II (7.75 ± 7.81% for TMVR and 6.91 ± 11.68% for SMVR) and Society of Thoracic Surgeons (STS) scores (8.34 ± 7.35% for TMVR and 8.48 ± 12.8% for SMVR). All patients had at least moderate MR, with a mean transmitral gradient of 3.2 ± 2.1 mm Hg for TMVR and 6.3 ± 4.1 mm Hg for SMVR. Baseline LVEF was lower in patients who underwent TMVR (42 ± 13%) compared with patients who underwent SMVR (57 ± 10%), whereas the LV end-diastolic volume (LVEDV) was similar between the 2 groups (146 ± 43 mL for TMVR and 134 ± 48 mL for SMVR), as well as the systolic pulmonary artery pressure (45 ± 11 mm Hg for TMVR and 43 ± 13 mm Hg for SMVR).Patients who underwent TMVR had lower tricuspid annular plane systolic excursion (TAPSE) values (18 ± 4 mm compared with 21 ± 3 mm in patients who underwent SMVR). They were more frequently affected by moderate-to-severe tricuspid regurgitation (TR) (53% for TMVR vs 8% for SMVR). Complete baseline demographic and echocardiographic data are available in Tables 1 and 2.

**Table 1:** Baseline demographic data

Variables	SMVR n (%) or mean ± SD	TMVR n (%) or mean ± SD	*P* value
Female	30 (60)	16 (53)	.559
Age, y	70 ± 10	75 ± 7	.023
BMI	25 ± 4	26 ± 5	.681
Arterial hypertension	34 (69)	26 (87)	.081
Diabetes mellitus type II	11 (22)	7 (23)	.928
COPD	5 (10)	6 (20)	.222
Creatinine, mg/dL	1 ± 1	1 ± 0.5	.787
Glomerular filtration rate, mL/min	57 ± 27	45 ± 19	.026
Coronary artery disease	13 (27)	9 (30)	.738
Previous MI	6 (12)	7 (23)	.197
Previous myocardial revascularization			
PCI	4 (8)	10 (33)	.004
CABG	−	5 (17)	.003
Previous valve surgery			
Aortic	−	8 (27)	<.001
Mitral	−	1 (3)	.194
Previous stroke	2 (4)	1 (3)	.866
EuroSCORE II (%)	6.91 ± 11.68	7.75 ± 7.81	.734
STS score (%)	8.48 ± 12.08	8.34 ± 7.35	.955
Atrial fibrillation	26 (53)	17 (57)	.755
ICD/PPM	−	14 (47)	<.001
HD	3 (6)	−	.198
Previous hospitalization for HF	25 (51)	22 (73)	.050
Previous hospitalization for HF number	0.6 ± 0.7	1 ± 1	.002
NYHA class			
II	20 (40)	18 (60)	
III	16 (32)	10 (33)	.054
IV	14 (28)	2 (7)	

*SMVR*, Surgical mitral valve replacement; *TMVR*, transcatheter mitral valve replacement; *SD*, standard deviation; *BMI*, body mass index; *COPD*, chronic obstructive pulmonary disease; *MI*, myocardial infarction; *PCI*, percutaneous coronary intervention; *CABG*, coronary artery bypass graft; *EuroSCORE*, European System for Cardiac Operative Risk Evaluation; *STS*, Society of Thoracic Surgeons; *ICD*, implantable cardiac defibrillator; *PPM*, permanent pacemaker; *HD*, hemodialysis; *HF*, heart failure; *NYHA*, New York Heart Association.

**Table 2:** Baseline echocardiographic parameters

Variables	SMVR n (%) or mean ± SD	TMVR n (%) or mean ± SD	*P* value
MR etiology			
Primary	29 (85)	6 (20)	
Secondary	4 (13)	20 (67)	<.001
Mixed	1 (3)	4 (13)	
MR grade			
Moderate	5	3 (10)	.5
Severe	45	27 (90)	
Aortic stenosis grade			
No/trivial	22 (78)	23 (77)	
Mild	2 (7)	7 (23)	.134
Moderate	1 (4)	−	
Severe	3 (11)	−	
Aortic regurgitation grade			
No/trivial	12 (41)	2 (8)	
Mild	8 (28)	16 (70)	.012
Moderate	8 (28)	5 (22)	
Severe	1 (3)	−	
MAC	11 (32)	6 (20)	.264
EROA (PISA), cm^2^	0.5 ± 0.2	0.4 ± 0.1	.058
Vena contracta, mm	7 ± 3	7 ± 2	.930
Mean mitral transvalvular gradient, mm Hg	6.28 ± 4.11	3.18 ± 2.11	.009
LV end-diastolic volume, mL	133 ± 48	146 ± 43	.369
LV end-systolic volume, mL	58 ± 32	78 ± 37	.103
LV ejection fraction, %	57 ± 10	42 ± 13	<.001
IVSd, mm	9 ± 4	11 ± 2	.074
LA volume, mL	114 ± 45	116 ± 43	.898
Tricuspid regurgitation grade			
No/trivial	5 (16)	−	
Mild	14 (42)	14 (47)	.048
Moderate	14 (42)	13 (43)	
Severe	−	3 (10)	
TAPSE, mm	21 ± 3	18 ± 4	.014
RV function			
Normal	27 (94)	21 (84)	
Mildly impaired	1 (3)	2 (8)	.283
Moderately impaired	−	2 (8)	
Severely impaired	1 (3)	−	
sPAP, mm Hg	43 ± 13	45 ± 11	.601

*SMVR*, Surgical mitral valve replacement; *TMVR*, transcatheter mitral valve replacement; *SD*, standard deviation; *MR*, mitral regurgitation; *MAC*, mitral annular calcification; *EROA*, effective regurgitant orifice area; *PISA*, proximal isovelocity surface area; *LV*, left ventricle; *IVSd*, interventricular septum thickness in end-diastole; *LA*, left atrial; *TAPSE*, tricuspid annular plane excursion; *RV*, right ventricle; *sPAP*, systolic pulmonary arterial pressure.

We had 1 intraoperative death in the SMVR group (the patient was critically ill and in cardiogenic shock at the time of surgery, with highly reduced LVEF). In contrast, all patients who underwent TMVR survived the procedure without any intraprocedural valve embolization, LV outflow tract obstruction, need for valve retrieval, access-site complication, or conversion to open heart surgery. We observed shorter postoperative lengths of stay in the intensive care unit (ICU) (*P* = .145) and a lower OT intubation time (*P* = .022) in the TMVR group (Table 3).

**Table 3:** Postoperative clinical outcomes

Variables	SMVR n (%) or mean ± SD	TMVR n (%) or mean ± SD	*P* value
ICU, d	6 ± 12	4 ± 6	.145
OT, h	15 ± 21	6 ± 5	.022
NYHA at discharge			
I	20 (48)	22 (76)	
II	20 (48)	7 (24)	.045
III	2 (4)	−	
All cause death (within 30 d)	9 (18)	1 (3)	.055
CV death (within 30 d)	9 (18)	1 (3)	.055
Surgical revision for bleeding	5 (10)	1 (3)	.273
Bleeding			
No	45 (90)	29 (97)	
Minor	−	−	
Major	5 (10)	1 (3)	.273
Extensive	−	−	
Life-threatening	−	−	
Acute MI	4 (8)	−	.110
CVVH	2 (5)	1 (3)	.780
Chronic dialysis	1 (2)	−	.403
Conduction disturbances	7 (17)	3 (10)	.420
New-onset atrial fibrillation			
No	32 (74)	29 (97)	
Paroxysmal	6 (14)	−	.035
Persistent	5 (12)	1 (3)	
Pericardial effusion			
No	23 (70)	16 (76)	
Minor	5 (15)	4 (19)	.487
Major	5 (15)	1 (5)	
Pleural effusion			
No	23 (49)	15 (52)	
Minor	15 (32)	11 (38)	.578
Major	9 (19)	3 (10)	
Sepsis	−	2 (7)	.090
Hospitalization from surgery, d	10 ± 8	10 ± 9	.980
Discharge			
Home	4 (8)	17 (57)	
Death	9 (18)	1 (2)	<.001
Rehabilitation or other unit	37 (74)	12 (41)	
Other ICU	−	−	

*SMVR*, Surgical mitral valve replacement; *TMVR*, transcatheter mitral valve replacement; *SD*, standard deviation; *ICU*, intensive care unit; *OT*, orotracheal intubatione; *NYHA*, New York Heart Association; *CV*, cardiovascular; *MI*, myocardial infarction; *CVVH*, continuous venovenous hemofiltration.

The 30-day all-cause mortality was 3% (1) and 18% (9), for TMVR and SMVR, respectively. Six of the patients who underwent SMVR who died within the first 30 days underwent surgery for active endocarditis and one was initially rejected for TMVR and rescheduled for surgical treatment despite high preoperative risk scores.

The 30-day CV mortality for patients who underwent TMVR was lower than for patients who underwent SMVR (3.3% vs 18% respectively, *P* = .055). Although not statistically significant, patients who underwent TMVR also experienced fewer surgical revisions for bleeding than patients who underwent SMVR (3.3% and 10%, respectively, *P* = .273). The onset of postoperative atrial fibrillation (AF) was greater in the SMVR group (11.6% vs 3.3% in the TMVR group, *P* = .035). Two patients in the SMVR group required continuous venovenous hemodialysis compared with one in the TMVR group (*P* = .780). In addition, patients who underwent TMVR were more frequently discharged home (*P* < .001). At discharge, 95.2% of patients in the SMVR group were in New York Heart Association (NYHA) class I-II, and 4.8% were in NYHA class III. All patients in the TMVR group were in NYHA class I-II (*P* = .045). No patients in both groups had more than mild paravalvular leak (PVL), with a transvalvular gradient lower than baseline. In the TMVR group, we observed no changes in LVEF (*P* = .111) compared with baseline, whereas patients who underwent SMVR had a slight decrease in LVEF from 56 ± 9% to 51 ± 7% (*P* = .031). Moreover, LVEDV significantly decreased in both groups whereas left atrial volume decreased only in the SMVR group. After SMVR, TAPSE significantly reduced (Δpre-post 26 ± 18%, *P* < .001), whereas after TMVR it remained stable (Δpre-post −0.4 ± 27%, *P* = .515) as well as systolic pulmonary arterial pressure (SMVR Δpre-post 23 ± 22% *P* < .001; TMVR Δpre-post 10 ± 22% *P* = .047), but there was no difference in changes between the 2 groups (*P* = .163). Right ventricular (RV) function worsened more frequently after SMVR than TMVR (46% and 15%, respectively, *P* = .05). Similarly, we observed more patients with worsened TR in the SMVR group, but this finding was not statistically significant. Complete echocardiographic data and comparative data are shown in Table 4 and Table E1.

**Table 4:** Pre-postoperative and postoperative-FU echocardiographic variations

Variables	Preoperative vs postoperative variations				Postoperative vs FU variations			
	SMVR n (%) or mean ± SD	*P* value	TMVR n (%) or mean ± SD	*P* value	SMVR n (%) or mean ± SD	*P* value	TMVR n (%) or mean ± SD	*P* value
Preoperative mean MV gradient, mm Hg	6.30 ± 4.0	.056	3.10 ± 2.10	.135	4.12 ± 1.52	.335	3.7 ± 1	.003
Postoperative mean MV gradient, mm Hg	4.13 ± 0.9		3.83 ± 1.17		4.68 ± 1.6		2.78 ± 1.5	
Δ mean MV gradient, %	5 ± 60	.022	−70 ± 113	.022	−49.27 ± 164.3	.039	25.80 ± 35.90	.039
Preoperative LVEDV, mL	133 ± 55	.026	157 ± 40	<.001	109 ± 41	.270	114 ± 31	.550
Postoperative LVEDV, mL	107 ± 42		118 ± 28		98 ± 30		107 ± 23	
Δ LVEDV, %	14 ± 29	.401	22 ± 22	.401	1.72 ± 35.49	.918	0.28 ± 31.8	.918
Preoperative LVEF, %	56 ± 9	.031	43 ± 14	.111	51 ± 8	.202	47 ± 9	.695
Postoperative LVEF, %	51 ± 7		47 ± 9		54 ± 10		46 ± 8	
Δ LVEF, %	7 ± 19	.002	−16 ± 28	.002	6.99 ± 21.23	.262	−0.09 ± 16.83	.262
Preoperative LA volume, mL	140 ± 44	<.001	122 ± 46	.450	82 ± 35	.679	127 ± 35	.387
Postoperative LA volume, mL	84 ± 17		111 ± 44		88 ± 43		116 ± 33	
Δ LA volume, %	37 ± 14	.004	1 ± 41	.004	−17.07 ± 51.98	.329	3.42 ± 35.7	.329
Preoperative TAPSE, mm	21 ± 4	<.001	18 ± 4	.515	15 ± 4	.007	18 ± 3	.610
Postoperative TAPSE, mm	15 ± 3		17 ± 3		19 ± 5		17 ± 3	
Δ TAPSE, %	26 ± 18	.007	−0.4 ± 27	.007	−34.72 ± 39.33	.005	1.13 ± 16.9	.005
Preoperative sPAP, mm Hg	47 ± 12	<.001	45 ± 11	.047	32 ± 6	.673	37 ± 10	.230
Postoperative sPAP, mm Hg	35 ± 9		38 ± 9		33 ± 6		35 ± 7	
Δ sPAP, %	23 ± 22	.163	10 ± 32	.163	−6.36 ± 28.32	.295	2.61 ± 21.5	.295

*FU*, Follow-up; *SMVR*, surgical mitral valve replacement; *TMVR*, transcatheter mitral valve replacement; *SD*, standard deviation; *MV*, mitral valve; *LVEDV*, left ventricle end-diastolic volume; *LVEF*, left ventricle ejection fraction; *LA*, left atrial; *TAPSE*, tricuspid annular plane excursion; *sPAP*, systolic pulmonary arterial pressure.

**Figure 2:**
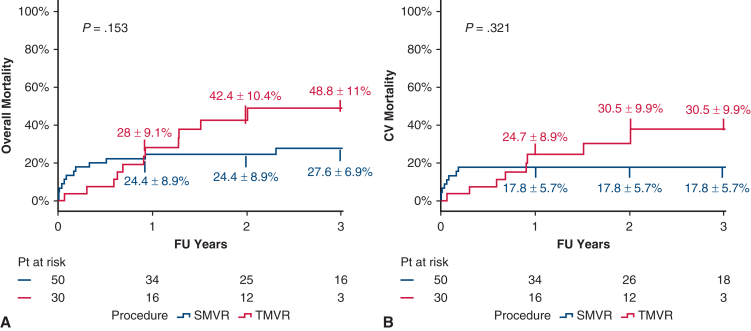
A, Kaplan-Meier cardiovascular mortality. B, Kaplan-Meier overall mortality. *SMVR*, Surgical mitral valve replacement; *TMVR*, transcatheter mitral valve replacement; *FU*, follow-up.

The median FU period was 1.92 (0.84-3.4) years. At FU, the overall mortality was 37.5% for SMVR and 50% for patients who underwent TMVR (*P* = .277). Moreover, CV mortality was 25% for SMVR and 33% for TMVR (*P* = .496). Cumulative overall mortality at 1 year and 2 years was 24.4 ± 6.4% for SMVR and 2 8 ± 9.1% and 42.4 ± 10.4% for TMVR (*P* = .153) (Figure 2, *A*). In addition, cumulative CV mortality at 1 year and 2 years was 17.8 ± 5.7% for SMVR and 24.7 ± 8.9%, and 30.5 ± 9.9% for TMVR (*P* = .321) (Figure 2, *B*). During the FU, 3 cases of endocarditis were observed in the SMVR group, 2 of which required redo surgery, and 1 case in the TMVR group, which was medically treated. No instances of prosthesis degeneration or thrombosis were observed during the FU period in both groups. However, there were 3 cases of deep sternal wound dehiscence in the SMVR group, leading to rehospitalization and minor surgical revision. At FU, most patients in both groups were in NYHA class I-II (93.3% in SMVR group and 86.7% in TMVR group, *P* = .752). The rehospitalization rate for HF was 20% in the SMVR group and 7.1% in the TMVR group (*P* = .352). Complete clinical FU data are reported in Table 5.

**Table 5:** FU clinical data

FU patient characteristics	SMVR, n (%)	TMVR, n (%)	*P* value
Overall mortality	18 (38)	15 (50)	.277
CV mortality	12 (25)	10 (33)	.496
Stroke	1 (3)	−	.475
NYHA class			
I	8 (27)	4 (27)	
II	20 (67)	9 (60)	.752
III	2 (6)	2 (13)	
Hospitalization for HF	5 (17)	1 (7)	.352
Reintervention	2 (7)	−	.306
MV surgery	2 (7)	−	.306
Endocarditis	3 (10)	1 (7)	.711
Wound dehiscence	3 (10)	−	<.001

*FU*, Follow-up; *SMVR*, surgical mitral valve replacement; *TMVR*, transcatheter mitral valve replacement; *CV*, cardiovascular; *NYHA*, New York Heart Association; *HF*, heart failure; *MV*, mitral valve.

At FU, the PVL rate was low in both groups and when it was present, it was mild. The mean transmitral gradient remained low in both groups. However, in the TMVR group, the mean gradient significantly decreased from discharge (2.8 ± 1.6 mm Hg to 3.7 ± 1 mm Hg, *P* = .003), whereas a similar trend was not observed in patients who underwent SMVR. The LVEF and LVEDV remained stable from discharge in both groups. However, TAPSE significantly improved in the SMVR group (Δpost-FU –34.7 ± 39.3%). RV function remained stable in both groups (k < 0 .2). There was a tendency for TR to worsen more in the SMVR group that in TMVR group (42% vs 15%, *P* = .101). Complete FU echocardiographic data and variations are reported in Table 4 and the Table E2.

At univariate analysis, the type of procedure (TMVR vs SMVR) did not have a significant impact on overall (hazard ratio [HR], 1.67; 95% confidence interval [CI], 0.82-3.41, *P* = .157) or CV survival (HR, 1.57; 95% CI, 0.66-3.73, *P* = .311). However, overall survival was affected by preoperative STS and EuroSCORE II, presence of diabetes, chronic obstructive pulmonary disease (COPD), previous percutaneous coronary intervention, number of hospitalizations for HF, and postoperative ICU length of stay. CV mortality was influenced by STS and EuroSCORE II, eGFR, previous percutaneous coronary intervention, COPD, baseline NYHA class, number of previous hospitalizations for HF, postoperative ICU length of stay, and OT intubation time (Table E3). These parameters were included in multivariable analysis, where COPD (HR, 3.15; 95% CI, 1.27-7.83; *P* = .013) and ICU length of stay (HR, 1.07; 95% CI, 1.03-1.10, *P* < .001) remained significant for overall survival, and the number of previous HF hospitalizations was the only parameter influencing CV survival (HR, 1.55; 95% CI, 1.04-2.30, *P* = .030). When adjusted for eGFR, preoperative TR grade, TAPSE, HF hospitalization, and MR mechanism, the procedure did not influence either CV or overall mortality.

## Discussion

The comparison of outcomes between SMVR and TMVR represents a new area of research. Up to now, only Ziegelmueller and colleagues^13^ have compared the 30-day clinical results of intermediate-risk patients who underwent SMVR and TMVR with the Tendyne System. Our study is the first to compare clinical and echocardiographic data at a median 2-year FU in a high-risk population.

On the basis of our experience, the type of procedure did not have any impact on midterm overall and CV survival. In addition, postoperative complications were similar between groups, with similar hospitalization lengths but a greater rate of home discharge in the TMVR population. At midterm FU, transmitral gradients were lower in the TMVR group, with a similar rate of mild PVL and LV remodeling. Moreover, apical access during TMVR did not affect early LV function, as demonstrated by unimpaired LVEF at discharge.

Univariate stepwise logistic regression analysis showed that the type of procedure did not affect survival at FU, even though 30-day mortality was greater in the SMVR population. Multivariable analysis confirmed that the only factors influencing survival were COPD and ICU length of stay for overall survival and previous hospitalization for HF for CV survival.

TMVR mortality is similar to that reported by other groups,^13^ whereas SMVR mortality is greater than previously reported elsewhere.^4^^,^^13^^,^^17^ Despite a relatively young age, the high surgical risk (indicated by STS and EuroSCORE II >7%) could explain this finding. Moreover, we observed a 10% surgical revision for bleeding and postoperative myocardial infarction (troponin increase after 72 hours and/or ST change on electrocardiogram) occurred in 8% of patients. These complications might have influenced early survival. The 30-day mortality rate observed in the SMVR cohort was also affected by the high rate of patients who were referred for endocarditis and many presenting with cardiogenic shock. Indeed, almost all deaths were observed in these patients. If we excluded these patients, we would have similar mortality between SMVR and TMVR groups. In presence of endocarditis, TMVR is not the preferred option because complete removal of infected tissue would be recommended; however, it has been previously described in literature with success^5^ and could be considered in extremely selected situations.

At midterm FU, the survival advantage initially offered by TMVR over SMVR is lost. This might be explained by the fact that early mortality is mainly influenced by hemodynamic instability and acute CV decompensation, which were more frequently found in patients who underwent SMVR. In the TMVR group, the number of previous hospitalizations for HF was greater than in the SMVR group, which might be attributable to concomitant chronic HF in patients who underwent TMVR.

On the basis of our findings, we might suggest that TMVR should be preferred over SMVR in this subset of patients. However, in cases of hemodynamic instability and urgent treatment, TMVR might not be feasible, as it requires specific preprocedural planning and has a high rejection rate for specific anatomical criteria.^11^

At FU, overall mortality was greater than CV mortality in both populations, indicating that survival is primarily affected by noncardiac medical conditions, especially renal function. It is interesting to note that the majority of patients who underwent TMVR presented a functional or mixed MR, which is usually associated with worst outcome in comparison with degenerative one. Despite this, the CV mortality in our population was acceptable, and at any rate survival was mostly influenced by noncardiac risk factors. The functional status showed significant improvement at discharge and FU in both populations, thanks to the complete resolution of MR, along with low transvalvular gradients and a low rate of device-related complications. We found no significant difference in postoperative complications between the groups. In contrast to other studies,^11^^,^^13^ we observed only a slight increase in the rate of major bleeding and surgical revision for bleeding after SMVR, but this was not statistically significant. Other groups have reported greater rates of complications related to apical access^9^^,^^11^^,^^13^ in comparison with our experience; this may be attributed to the optimal management of apical access during the procedure by our experienced team.^18^, ^19^, ^20^, ^21^ We did not observe any perioperative strokes in either group, whereas one late ischemic stroke was recorded during FU in a patient who underwent SMVR. The low occurrence of neurologic injuries in our TMVR population may be attributable to effective oral anticoagulation management with vitamin K antagonists and the low rate of new postoperative AF. After undergoing TMVR, patients experienced a lower occurrence of AF compared with SMVR. This could be attributed to the reduced systemic inflammatory response resulting from avoiding cardiopulmonary bypass and fluctuations in intravascular volume. Similar findings have been reported in previous studies comparing surgical aortic valve replacement and transcatheter aortic valve replacement (TAVR),^22^^,^^23^ as well as on-pump and off-pump coronary artery bypass grafting,^24^^,^^25^ providing support for this hypothesis.

In contrast to Ziegelmueller and colleagues,^13^ we did not observe any differences in the length of hospital stays between the 2 groups despite patients who underwent TMVR being more frequently discharged home. The longer in-hospital stay for patients who underwent TMVR may be attributable to facilitating their discharge to home. However, the fact that patients who underwent TMVR were more often discharged home compared with the need for rehabilitation in the case of SMVR suggests a quicker recovery in the transcatheter group.

After TMVR, there was no significant change in postoperative LVEF. In contrast, as previously reported, postoperative LVEF significantly decreased after SMVR.^26^^,^^27^ This suggests that with experienced teams, TMVR using apical access is a safe approach that LV function, as previously described for TAVR.^28^ In addition, TMVR allows the subvalvular mitral apparatus to remain in place, whereas in SMVR, the anterior leaflet and its subvalvular apparatus typically are removed. RV function significantly worsened after SMVR but improved during FU. This trend was not observed in the TMVR group, where TAPSE and RV function remained stable at discharge and during FU. The absence of cardiopulmonary bypass and cardioplegic arrest in the TMVR group may have contributed to this finding. On the basis of this evidence, it may be advisable to choose TMVR over SMVR when patients with moderate-to-severe RV dysfunction require mitral surgery, as acute RV failure negatively affects early and long-term survival after MV surgery.^29^^,^^30^ We noticed a greater decrease in transmitral gradients after surgery in the SMVR group compared with the TMVR group. This may be because the surgical group had more cases of concomitant MS with greater preoperative gradients. However, during FU, we observed a significant further decrease in the TMVR group, which was not seen in the surgical group (TMVR 26% decrease, SMVR 49% increase, *P* = .039). This difference could potentially have a positive effect on LV remodeling over a more extended follow-up period.

According to our results, TMVR with the Tendyne System provides comparable clinical and echocardiographic midterm results respect to SMVR in a high-risk population. General patient conditions had a greater impact on mortality than the procedure itself, as indicated by the low early mortality rate. Treating patients with a lower risk profile may result in better midterm outcomes and could potentially shift the paradigm toward TMVR, similar to the transition observed with TAVR in the treatment of aortic valve stenosis.

### Limitations of the Study

The main limitation of this study is its retrospective nature and the relatively small sample size, which prevented us from conducting a propensity-matched analysis. However, by using a stepwise logistic approach to analyze the procedure's effect on midterm outcomes, we were able to partially compensate for this bias. In addition, both univariate and multivariate analyses showed that preoperative variables did not influence the outcome, which may help to reduce selection bias.

## Conclusions

We have observed that both SMVR and TMVR resulted in similar midterm clinical and echocardiographic outcomes in a high-risk population. However, TMVR showed lower early postoperative complication rates and faster recovery in frail, high-risk patients. Therefore, for selected high-risk patients, TMVR could be a valuable alternative to surgery. To expand indications, technical and procedural improvements^27^, ^28^, ^29^ and faster and easier screening pathways are necessary.^30^ Furthermore, results of clinical randomized trials comparing SMVR and TMVR, such as the SUMMIT trial (NCT03433274), are needed to confirm this initial evidence.

## Conflict of Interest Statement

A.C. is a proctor for Abbott Cardiovascular; all other authors reported no conflicts of interest.

The *Journal* policy requires editors and reviewers to disclose conflicts of interest and to decline handling or reviewing manuscripts for which they may have a conflict of interest. The editors and reviewers of this article have no conflicts of interest.

## Figures and Tables

**Table E1:** Postoperative echocardiographic parameters

Variables	SMVR n (%) or mean ± SD	TMVR n (%) or mean ± SD	*P* value
Mean MV gradient, mm Hg	4.51 ± 1.58	3.69 ± 1.14	.023
LV end diastolic volume, mL	104 ± 39	118 ± 27	.160
LV ejection fraction, %	52 ± 7	47 ± 9	.031
LA volume, mL	79 ± 28	112 ± 44	.008
sPAP, mm Hg	33 ± 8	38 ± 10	.089
TAPSE, mm	15 ± 3	18 ± 3	.007
PVL	3 (9)	4 (14)	.559
PVL grade			
No	30 (90)	25 (86)	.559
Mild	3 (10)	4 (4)	
RV function			
Normal	18 (58)	18 (75)	
Mildly reduced	12 (39)	6 (25)	.342
Moderately reduced	−	−	
Several	1 (3)	−	
TR grade			
No/trivial	7 (24)	2 (8)	
Mild	14 (48)	14 (58)	.334
Moderate	7 (24)	8 (34)	
Severe	1 (4)		

*SMVR*, Surgical mitral valve replacement; *TMVR*, transcatheter mitral valve replacement; *SD*, standard deviation; *MV*, mitral valve; *LV*, left ventricle; *LA*, left atrial; *sPAP*, systolic pulmonary arterial pressure; *TAPSE*, tricuspid annular plane excursion; *PVL*, paravalvular leak; *RV*, right ventricle; *TR*, tricuspid regurgitation.

**Table E2:** FU echocardiographic parameters

FU patient characteristics	SMVR n (%) or mean ± SD	TMVR n (%) or mean ± SD	*P* value
Mean MV gradient, mm Hg	4.64 ± 1.56	2.78 ± 1.59	<.001
LVOT gradient, mm Hg	1.7 ± 0.6	1.5 ± 1.1	.768
LV end diastolic volume, mL	100 ± 27	113 ± 33	.234
LV end systolic volume, mL	43 ± 24	59 ± 29	.200
IVSd, mm	11 ± 2	9 ± 4	.128
LV ejection fraction, %	55 ± 10	45 ± 10	.001
LA volume, mL	92 ± 38	122 ± 36	.027
TAPSE, mm	18 ± 5	17 ± 3	.595
sPAP, mm Hg	32 ± 6	35 ± 8	.209
PVL	3 (13)	7 (29)	.177
PVL grade			
No	20 (87)	17 (71)	.177
Mild	3 (13)	7 (29)	
RV function			
Normal	15 (68)	19 (80)	
Mildly reduced	3 (14)	3 (13)	.591
Moderately reduced	4 (18)	2 (7)	
TR grade			
No/trivial	−	−	
Mild	15 (71)	12 (60)	
Moderate	4 (20)	7 (35)	.691
Severe	2 (9)	1 (5)	

*FU*, Follow-up; *SMVR*, surgical mitral valve replacement; *TMVR*, transcatheter mitral valve replacement; *SD*, standard deviation; *MV*, mitral valve; *LVOT*, left ventricular outflow tract; *LV*, left ventricle; *IVSd*, interventricular septum thickness in end-diastole; *LA*, left atrial; *TAPSE*, tricuspid annular plane excursion; *sPAP*, systolic pulmonary arterial pressure; *PVL*, paravalvular leak; *RV*, right ventricle; *TR*, tricuspid regurgitation.

**Table E3:** Overall mortality and CV mortality univariate analysis

Variables	Overall mortality			CV mortality		
	HR	95% CI	*P* value	HR	95% CI	*P* value
Age, y	1.02	0.10-1.07	.206	1.04	0.98-1.09	.177
BMI	0.98	0.90-1.08	.705	0.98	0.89-1.09	.717
STS score, %	1.04	1.01-1.07	.002	1.05	1.02-1.08	<.001
EuroSCORE II, %	1.04	1.00-1.07	.015	1.05	1.02-1.08	.001
Glomerular filtration rate, mL/min	0.99	0.97-1.00	.135	0.97	0.95-0.99	.006
Diabetes mellitus type II	2.26	1.10-4.63	.026	2.24	0.93-5.42	.074
Hypertension	1.50	0.61-3.66	.377	2.06	0.60-7.05	.251
Previous MI	1.95	0.87-4.36	.106	2.34	0.90-6.09	.082
Previous PCI	2.12	0.97-4.62	.058	2.67	1.06-6.71	.037
Previous CABG	1.86	0.56-6.17	.310	2.79	0.81-9.59	.103
COPD	3.45	1.50-7.95	.004	2.77	0.99-7.78	.053
ICD/PPM	1.32	0.57-3.07	.525	1.86	0.72-4.86	.203
Previous hospitalization for HF	1.77	0.83-3.75	.139	4.98	1.45-17.13	.011
Previous hospitalization for HF number	1.37	1.06-1.78	.017	1.65	1.25-2.18	<.001
NYHA class	1.23	0.82-1.87	.321	1.84	1.11-3.06	.018
MAC	1.87	0.85-4.09	.118	1.98	0.76-5.12	.160
LV end-diastolic volume, mL	1.00	0.99-1.01	.845	1.00	0.99-1.01	1.974
LV ejection fraction, %	0.97	0.95-1.00	.067	0.98	0.94-1.01	.191
Tricuspid regurgitation grade	1.35	0.79-2.29	.270	1.57	0.81-3.03	.178
TAPSE, mm	0.95	0.86-1.05	.282	0.90	0.79-1.02	.090
RV function	1.52	0.78-2.95	.218	1.83	0.94-3.55	.076
sPAP, mm Hg	0.99	0.95-1.03	.541	0.96	0.92-1.01	.107
ICU, d	1.04	1.02-1.07	<.001	1.04	1.01-1.06	.006

*CV*, Cardiovascular; *BMI*, body mass index; *STS*, Society of Thoracic Surgeons; *EuroSCORE*, European System for Cardiac Operative Risk Evaluation; *MI*, myocardial infarction; *PCI*, percutaneous coronary intervention; *CABG*, coronary artery bypass graft; *COPD*, chronic obstructive pulmonary disease; *ICD*, implantable cardiac defibrillator; *PPM*, permanent pacemaker; *HF*, heart failure; *NYHA*, New York Heart Association; *MAC*, mitral annular calcification; *LV*, left ventricle; *TAPSE*, tricuspid annular plane excursion; *RV*, right ventricle; *sPAP*, systolic pulmonary arterial pressure; *ICU*, intensive care unit.
